# Microfungal oasis in an oligotrophic desert: diversity patterns and community structure in three freshwater systems of Cuatro Ciénegas, Mexico

**DOI:** 10.7717/peerj.2064

**Published:** 2016-06-02

**Authors:** Patricia Velez, Jaime Gasca-Pineda, Edmundo Rosique-Gil, Luis E. Eguiarte, Laura Espinosa-Asuar, Valeria Souza

**Affiliations:** 1 Departamento de Ecología Evolutiva, Instituto de Ecología, Universidad Nacional Autónoma de México, Mexico City, Mexico; 2 División Académica de Ciencias Biológicas, Universidad Juárez Autónoma de Tabasco, Villahermosa, Tabasco, Mexico

**Keywords:** Ascomycota, Submerged wood baits, Biodiversity, Facultative aquatic fungi, Oasis mycobiota, Plant debris

## Abstract

The Cuatro Ciénegas Basin (CCB) comprises several oligotrophic aquatic ecosystems limited by phosphorus. These aquatic systems are dominated by a high prokaryotic diversity, shaped by the stress of low nutrient supplies and interspecific competition. Although fungi constitute a diverse and important component of microbial diversity, the microfungal diversity in the CCB remains to be unveiled. With the aim to explore microfungal diversity and ecological patterns in this area, we present the first investigation analyzing cultivable taxa from sediment and water, as well as lignocellulolytic taxa obtained from incubated submerged plant debris, and wood panels in three contrasting freshwater systems in the CCB: Churince, Becerra and Pozas Rojas. We chose a culture-based approach to analyze sediment and water samples in order to obtain fungal cultures, providing opportunities for *a posteriori* studies, and the possibility of *ex situ* preservation of the diversity. We evaluated sequence data from the nuclear ribosomal internal transcribed spacer including the 5.8 rDNA region for 126 isolates, revealing 37 OTUs. These OTUs were phylogenetically affiliated to several genera in the fungal phyla: Zygomycota, Basidiomycota, and Ascomycota. We recorded two OTUs with saline affinity, agreeing with previous findings on the prokaryotic communities with ancestral marine resemblances. All the studied systems showed moderate diversity levels, however discrepancies among the diversity indexes were observed, due to the occurrence of abundant taxa in the samples. Our results indicated that lignocellulolytic microfungal communities are dominated by transient fungal taxa, as resident species were not recorded perhaps as a result of the long-term strong competition with the highly adapted prokaryotic community. Moreover, the obtained microfungal taxa occurred mostly on the resident plant debris, rather than submerged wood panels, perhaps as a result of the high adaptation to specific environmental conditions. In conclusion, the CCB possess a moderate taxonomical diversity compared to other arid environments, probably as a result of high selective pressures. Nonetheless, due to high spatial and temporal heterogeneity, the functional fungal diversity was considerable as predicted by the intermediate disturbance hypothesis. Decisively, the assessment of microfungal diversity freshwater systems is relevant, since this ecological group of microorganisms represents an important indicator of trophic complexity and biotic interactions among microbial communities, having important implications for understanding eukaryotic survival at the oligotrophic limit for life.

## Introduction

Microorganisms represent the invisible majority of biodiversity, comprising a great portion of the genetic diversity on Earth (Whitman, Coleman & Wiebe, 1998). These organisms influence a large number of important ecosystem processes, such as nutrient acquisition, biogeochemical processes, and soil formation (Hogberg et al., 2001; Kowalchuk & Stephen, 2001; Sprent, 2001; Rillig & Mummey, 2006). Therefore, they act as important regulators of plant productivity, community dynamics and diversity in nutrient-poor and extreme ecosystems (Van Der Heijden, Bardgett & Van Straalen, 2008). Remarkably, fungi represent a large and diverse component of microbial diversity (Fierer et al., 2007).

Freshwater ecosystems have a primary role in the biosphere, supporting unique and complex ecological communities, which often define the structure and functioning of the surrounding terrestrial ecosystem (Bailey, Norris & Reynoldson, 2004). These systems are closely related to the riparian zone, which provides large inputs of woody and herbaceous debris, regulating the transfer of energy between both systems (Webster et al., 1999; Pusey & Arthington, 2003). Plant debris represents an essential source of energy for heterotrophs, yet it is not directly accessible to most freshwater invertebrates and bacteria since they lack the enzymes needed to degrade complex plant structural compounds, such as lignin and cellulose (Dudley & Anderson, 1982; Bärlocher & Porter, 1986; Wetzel, 1995; Romaní et al., 2006).

Microfungi in aquatic environments range from those entirely adapted to complete their life cycles in aquatic habitats, and are not found outside of the water (residents or truly aquatic), to those occurring in water incidentally by being washed or blown in (transients or facultative aquatic) (Shearer et al., 2007). Anyhow, both resident and transient aquatic microfungi constitute a key element of the freshwater microbial communities, since they play a vital role in the functioning of aquatic ecosystems, fulfilling crucial activities as part of food webs and in nutrient cycling (Zare-Maivan & Shearer, 1988a; Zare-Maivan & Shearer, 1988b; Shearer, 1993; Gessner & Chauvet, 1997; Wong et al., 1998; Buesing & Gessner, 2006; Vijaykrishna, Jeewon & Hyde, 2006).

The Cuatro Cienegas Basin (CCB) epitomizes an arid ultra-oligotrophic oasis and unique ecosystem, harboring the highest level of endemisms in North America (Abell et al., 2000; Elser et al., 2005; Souza et al., 2006; Souza et al., 2008; Desnues et al., 2008; Bonilla-Rosso et al., 2012). This arid biome includes diverse aquatic systems strongly limited by phosphorus, surrounded by halophilic and gypsophilic grassland (Elser et al., 2005; Elser et al., 2006; López-Lozano et al., 2012). Furthermore, aquatic systems within the CCB represent a priority site for conservation by the Nature Conservancy, the World Wildlife Fund, and the United Nations Educational, Scientific and Cultural Organization (UNESCO), and have been listed as a Wetland of International Importance within the international Ramsar Convention (Souza et al., 2012).

Previous studies have suggested that aquatic systems in CCB are dominated by a high diversity of prokaryotic taxa, shaped by the stress of low nutrient supplies and interspecific competition (Souza et al., 2012). Nevertheless, there is no information regarding the microfungal diversity inhabiting this unique environment. As studies analyzing the diversity and function of fungal microorganisms are needed for CCB, the aims of this study were: (i) to analyze the diversity patterns of filamentous microfungi from water and sediment samples, (ii) to describe the diversity of lignocellulolytic microfungi from submerged local plant debris and test blocks (iii) to determine ecological preferences of the obtained microfungal communities in three distinct spring fed pool systems in the endangered ultra-oligotrophic desert oasis of CCB. We chose a culture-based approach to analyze sediment and water samples in order to obtain fungal cultures, which provide opportunities for *a posteriori* physiological, genomic and ecological studies, and the possibility of *ex situ* preservation of the diversity.

We expected to record low taxonomical diversity of microfungi due to oligotrophic conditions of CCB and antagonistic interactions with the highly competitive prokaryotic community, as well as a strong geographic differentiation based on previous results on bacterial diversity and the topological characteristics of the study area (Elser et al., 2005; Escalante et al., 2008; Souza et al., 2012). Lastly, we assumed the dominance of highly adapted lignocellulolytic microfungal diversity occurring on local vegetation remains compared to submerged *Pinus* wood traps, as a result of a high specificity and adaptation to the local lignocellulose source, as previous work on the palaeoenvironmental history of CCB suggest that since ca. 11,000 years ago, woodlands are restricted to occur upslope (Minckley & Jackson, 2008).

## Materials and Methods

### Study site

This work was conducted in the CCB (26°50^′^N; 102°80^′^W), in the Chihuahuan desert, Coahuila State, Mexico. The climate is hot and arid, with two marked seasons. The first season from November to April is cold and dry, with a minimum and maximum temperature of 4 and 31 °C, respectively, and 51 mm of precipitation. The second season from May to October is hot, with a minimum and maximum temperature of 15 and 35 °C, respectively, and 155 mm of precipitation, which represents 60% of the annual total (SMN & CONAGUA, 2015). The soil is dominated by gypsisols and leptosols (Mckee, Jones & Long, 1990; IUSS Working Group WRB, 2007). Within the basin, a large number of springs, spring-fed streams and terminal evaporitic ponds form an inverse archipelago, in which aquatic systems are bounded by sparse desert vegetation, microbial crusts and salty soils (Minckley & Cole, 1968). The main vegetation types are grassland and desert scrub, dominated by the grass *Sporobolus airoides* and including element of Amaranthaceae, such as *Allenrolfea occidentalis*, Euphorbiaceae, including *Jatropha dioica*, and Zygophyllaceae, in particular *Larrea tridentata* (Perroni, García-Oliva & Souza, 2014).

We sampled three freshwater systems in the Cuatro Ciénegas Basin (CCB), Coahuila Mexico, within the Flora and Fauna Protection Area in Coahuila, Mexico under the administration of the Comisión Nacional de Áreas Naturales Protegidas (CONANP): Churince, Pozas Rojas and Becerra under the field permit FAUT-0230, emitted by Secretaría de Medio Ambiente y Recursos Naturales, Subsecretaría de Gestión para la Protección Ambiental, Dirección de Vida Silvestre. The Churince aquatic system (CH) comprises a spring and a shallow lagoon, connected by a river. It is rich in calcium and sulfates, but very poor in total phosphorous, P (Mckee, Jones & Long, 1990; Elser et al., 2005; Souza et al., 2006). The Pozas Rojas system (PR) represents a fluctuating environment, consisting of several small desiccation ponds with drastically fluctuating conditions (Rebollar et al., 2012). Former studies have reported that the microorganisms in PR are specialized in the degradation of a wide and complex array of metabolic byproducts as a result of antagonistic coevolution (Peimbert et al., 2012). Finally, Becerra (BE), the main stream in the wetland, is a stable warm inflow spring (34.7 °C) enriched in sulfate (Johannesson, Cortés & Kilroy, 2004; Escalante et al., 2008). Based on the analysis of terminal restriction fragment length polymorphisms (T-RFLP), the prokaryotic communities in the BE system have been demonstrated to resemble the adjacent CH system (Escalante et al., 2009).

### Sampling

**Figure 1:**
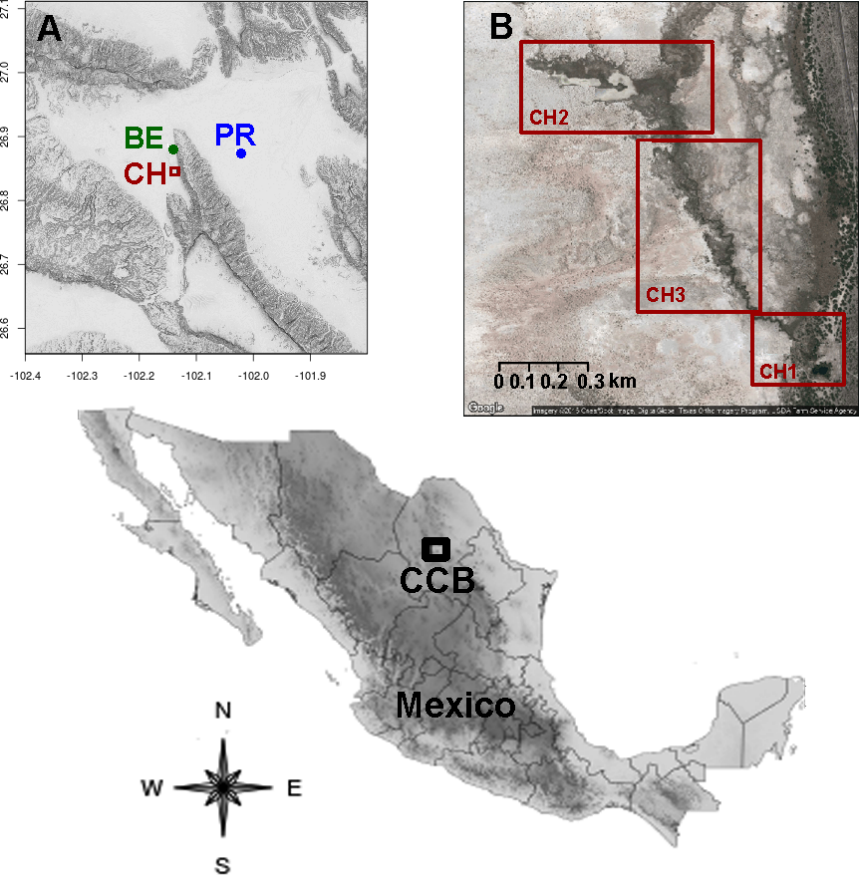
Location of the sampling sites in the Cuatro Ciénegas Basin, Coahuila, Mexico. (A) Geographical position of the studied freshwater systems within the CCB, and (B) detail of the sampling sites in the Churince system.

Based on the geographical proximity and abiotic characteristics, we characterized the sampling sites in five regions (Fig. 1, Table 1). We collected a total of 28 sediment and water samples in each site in June 2014 and March 2015. The water samples were collected from the surface, and the sediment samples at 10 cm depth in the margin of each analyzed freshwater system. Labeled sterile 50 mL Falcon^®^ tubes (Becton Dickinson, Cowley, Oxford, UK) were filled to the brim with samples and stored at 4 °C in the dark and transferred to the laboratory for further processing.

**Table 1:** Sampled ultra-oligotrophic freshwater systems in the Cuatro Ciénegas Basin.

Nomenclature	Hydrologic system	Coordinates
CH1	Churince	26°50′25.3″N, 102°8′2.9″W
	Churince	26°50′25.3″N, 102°8′3.7″W
	Churince	26°50′27.4″N, 102°8′5.2″W
CH2	Churince	26°50′33.4″N, 102°8′12.2″W
	Churince	26°50′36.2″N, 102°8′15″W
	Churince	26°50′37.5″N, 102°8′16.2″W
	Churince	26°50′40.6″N, 102°8′16.1″W
	Churince	26°50′42.8″N, 102°8′17.7″W
	Churince	26°50′47.3″N, 102°8′22.2″W
	Churince	26°50′53.5″N, 102°8′21.4″W
CH3	Churince	26°50′53″N, 102°8′29.7″W
	Churince	26°50′53.8″N, 102°8′30″W
	Churince	26°50′55.1″N, 102°8′31.9″W
	Churince	26°50′55.2″N, 102°8′31.6″W
	Churince	26°50′55.8″N, 102°8′35.7″W
BE	Becerra	26°52′40.99″N, 102°8′17.41″W
	Becerra	26°52′41.07″N, 102°8′18.07″W
	Becerra	26°52′41.67″N, 102°8′18.17″W
	Becerra	26°52′43.12″N, 102°8′17.46″W
	Becerra	26°52′41.82″N, 102°8′17.18″W
PR	Pozas Rojas	26°52′16.8″N, 102°1′11.3″W
	Pozas Rojas	26°52′16.7″N, 102°1′10″W
	Pozas Rojas	26°52′18.3″N, 102°1′12.3″W
	Pozas Rojas	26°52′18.7″N, 102°1′16.7″W
	Pozas Rojas	26°52′18.3″N, 102°1′16.5″W
	Pozas Rojas	26°52′17.9″N, 102°1′17.6″W
	Pozas Rojas	26°52′15.6″N, 102°1′16.9″W
	Pozas Rojas	26°52′16.1″N, 102°1′13.3″W

Additionally, to assess the diversity of lignocellulolytic fungi playing an active role in the degradation of plant remains in each system (Table 1), 25 wood panels were submerged for six months during the transition from wet to dry season (from September 2014 to March 2015), according to the baiting technique described by Jones (1971). Briefly, this technique consists in submerging test blocks in aquatic ecosystems, and after a determined period, test blocks are recovered, incubated and examined for fungal structures. A variety of timbers have been reported, yet for Mexican freshwater systems *Pinus* sp. baits have been successfully used (Chavarria et al., 2010; Rosique-Gil, Cifuentes-Blanco & González, 2008). The exposed wood panels and naturally occurring submerged plant remains from the autochthonous vegetation around the study sites were collected (for a total of 25 plant remains samples), placed in separate Zip-lock^®^ plastic bags, transported to the laboratory in a cooler containing ice to control biological activity, and processed within 24 h. Collected wood panels were washed in tap water (to remove microfauna, silt and mud from the surface) and placed in transparent plastic boxes containing moist paper towels in order to conform a moist chamber and induce the production of reproductive fungal structures. Local plant remain samples were similarly incubated in Zip-lock^®^ plastic bags with moist paper towels.

### *Ex situ* processing, fungal isolation, and identification

**Sediment and water samples**. Microfungi from water and sediment samples were isolated according to the dilution plate method (Warcup, 1960), using two culture media: Corn Meal Agar (Fluka Analytical, Sigma-Aldrich, St. Louis, MO, USA) and Potato Dextrose Agar (PDA; Fluka Analytical, Sigma-Aldrich, St. Louis, MO, USA), according to the manufacturer’s instructions. Dilution plates were prepared using 1 g of each sediment sample, and 1 mL of each water sample, at 10^−1^–10^−6^ dilutions in test tubes with sterilized distillated water. For the initial isolation of fungi, culture media were added with 0.5 g/mL of penicillin-G and 0.3 g/mL of streptomycin sulfate to prevent bacterial development. Two replicates for each sample were plated at a rate of 1 mL aliquot of each dilution series. Dilution plates were incubated for 7 days at 25 °C with a 12 h photoperiod. The plates were examined daily, and each colony that developed was subsequently transferred to a plate with PDA medium. We conducted a preliminary clustering of morphotypes based on the macroscopic morphology of colonies, yet the taxonomic assignment was based on the analysis of the internal transcribed spacer sequences of nuclear ribosomal DNA (ITS1-5.8S rDNA-ITS2), hereafter referred as ITS region.

**Lignocellulose material.** Wood panels (*Pinus* sp.) and plant remains (herbaceous and lignicolous substrata) were incubated in moist chambers according to the methodology described by Jones (1971). Incubation conditions were as follows: room temperature (22–26 °C) under natural daylight, and examined within 1 wk of collection and periodically over 6 mo under a stereo microscope (Discovery V8 Stereo, Carl Zeiss) for the presence of fungal structures. We chose a 6 mo incubation period based on: (1) the average incubation period reported on literature (i.e., Ho et al., 2002; Luo et al., 2004; Cai, Ji & Hyde, 2006; Rosique-Gil, Cifuentes-Blanco & González, 2008; Raja & Shearer, 2008) and, (2) previous experience with Mexican material, where a maximum 4 weeks incubation has been proved to be optimal for obtaining most of the lignocellulolytic fungal diversity (Rosique-Gil, Cifuentes-Blanco & González, 2008; Chavarria et al., 2010). After the production of reproductive structures, identification was based on the examination of the morphology of fungal reproductive structures using specialized literature (Boerema et al., 2004; Gräfenhan et al., 2011; Zhang et al., 2011; Luo & Zhuang, 2012; Wang et al., 2015; among others). For the morphological examination, squash mounts on glass slides of fungal structures in sterile water were prepared, and fungal structures (ascomata, paraphyses, asci, ascospores, conidiophores, conidia, phialides, etc.) were measured and photographed using a Carl Zeiss Axiostar Plus transmitted light microscope equipped with the Zeiss ZEN microscope software. Measurements were made of material mounted in water.

### DNA extraction, amplification and sequencing

Pure fungal isolates obtained from sediment and water samples were transferred to Potato Dextrose liquid medium and incubated in the dark, at 25 °C until adequate growth occurred (regularly for 6 days). Mycelium was collected and DNA was isolated using the technique described by Doyle & Doyle (1987) The ITS rDNA region was amplified and sequenced using primers ITS1 and ITS4 as previously described (White et al., 1990). Sequencing reactions were performed by the High Throughput Genomics Center Facility, University of Washington and by the Macrogen Inc., South Korea. Cultures and total DNA were deposited in the culture collection of the Laboratorio de Evolución Molecular y Experimental, Instituto de Ecología, Universidad Nacional Autónoma de México, headed by Dr. Valeria Souza, and are available for research upon request.

### Bioinformatics analyses

The quality assessment, as well as the assembly of the forward and the reverse sequences was performed using the finishing tool Consed version 27.0 (Ewing & Green, 1998; Ewing et al., 1998; Gordon, Desmarais & Green, 2001). The taxonomic assignment of the 126 sequences obtained from fungi isolated from sediment and water samples, was conducted via two methodologies in order to obtain the highest accuracy. First, the assembled sequences were clustered into operational taxonomic units (OTUs) with the bioinformatic program Cd-hit, and then assigned to a taxonomic group through the comparison to UNITE database (Abarenkov et al., 2010; Koljalg et al., 2005; Li & Godzik, 2006). Second, the assembled sequences were clustered using Blastclust (Rämä et al., 2014), (accessible at: http://toolkit.tuebingen.mpg.de/blastclust), and compared to the Genbank Data Base through a BLAST search in order to obtain at least one reference isolate for each CCB isolate. Only hit sequences with a minimum cover of 94% of the sequence length were considered, preferably including accessions associated with voucher strains. Environmental sequences/samples in the database were excluded. For each OTU, several hits from different studies were considered, preferably from published studies. A list with the Genbank Data Base accession numbers of reference sequences, as well as the analyzed the CCB sequences accession numbers, isolates correspondence, and OTU designation are reported on supplementary materials Table S1. For both Cd-hit and Blastclust, the homology for defining cultivable fungal OTUs was set as proposed by Millberg, Boberg & Stenlid (2015), with a sequence similarity cut-off value of 98–100% for presumed species, 94–97% for genus level and 80–93% for order level. For conflicting hits, the lowest common rank level was used for taxonomic assignment (Peršoh et al., 2010; Table S1).

### Phylogenetic analyses

We performed Neighbor-Joining (NJ), Maximum Likelihood (ML) and Bayesian phylogenetic reconstructions to further test the accuracy of the bioinformatics analyses for OTUs clustering and taxonomic designation in an evolutive context. The NJ analysis was carried out using the software Ninja version 1.2.1 (Wheeler, 2009). The ML tree was done using PhyML version 3.0 (Guindon et al., 2010) using the BEST tree topology search and 500 non-parametric bootstrap replicates for branch support. The Bayesian reconstruction was computed with MrBayes version 3.2.2 (Ronquist et al., 2012), we conducted four parallel runs with one cold chain and four heated chains each for 10,000,000 generations starting with a random tree and sampling every 1,000 steps. The substitution model used for the ML and Bayesian analyses was GTR+I+G, obtained by jModel Test version 2.1.7 (Darriba et al., 2012), the model was selected using both AIC and BIC criteria. The trees were visualized using FigTree version 1.4.2 (Rambaut, 2009).

### Community structure

We estimated the diversity within (*α*) and between (*β*) the studied freshwater systems, using the packages vegan version 2.3-1 (Oksanen et al., 2015) and ade4 version 1.7.2 (Dray & Dufour, 2007) implemented in R version 3.2.0 (R Development Core Team, 2015). We calculated several indexes displaying distinctive traits of diversity and variances among systems. To evaluate the diversity considering OTUs dominance, we estimated the Shannon–Weaver (*H*′) and Simpson (*D*) indexes, whereas to weight for the number of species, we used the Pielou’s (*J*) and Simpson’s (*E*) evenness indexes. Additionally, we included the *α* parameter of Fisher’s log-series (Fisher, Corbet & Williams, 1943). This measure of diversity behaves asymptotically equal to the rarefied species richness, i.e., it is less biased by differences in the number of isolates per sample. To evaluate the diversity between the freshwater systems based on a rarefaction approach, we used the series of Rényi and Tsallis diversities (Hurlbert, 1971; Hill, 1973). We plotted the upper, lower and median values of rarefied diversity of the overall data set and compared it to the values obtained for each site. To evaluate the differences in OTU composition between springs we calculated the Bray-Curtis and Jaccard dissimilarity matrices (Gower & Legendre, 1986). The Bray-Curtis considers the abundance of OTUs between systems, whereas Jaccard dissimilarity uses a binary approach, just considering the presence or absence of certain OTU in a site.

Since OTUs richness could be influenced by differences in the number of dilution plates per sampling unit (freshwater systems), we implemented different methods based on rarefaction to evaluate the effect of such differences in the number of isolates sampled in each system. We estimated an accumulation curve for the number of OTUs adding the sites at random using 100 permutations; subsequently we used an individual approximation considering the OTUs recovered given the samples (total number of sequences) (Colwell et al., 2012).

Additionally, we performed a detrended correspondence analysis (DCA) in order to evaluate the OTUs dissimilarities between sites. This ordination method rescales the axes to equal variances of species scores, and down-weight the influence of rare species. Furthermore, this approximation allowed us to evaluate the relative contribution of OTUs to the overall composition differences among sites (Hill & Gauch, 1980; Oksanen & Minchin, 1997). All graphics and statistical analyses were carried out using R version 3.2.0 (R Development Core Team, 2015). Maps were generated using the elevation layers from Instituto Nacional de Estadística y Geografía (INEGI, 2013) with the packages sp (Pebesma & Bivand, 2005) and maptools (Bivand & Lewin-Koh, 2015). Churince River was ploted using gmap from package dismo (Hijmans et al., 2015).

## Results

### Sediment and water samples

**Figure 2:**
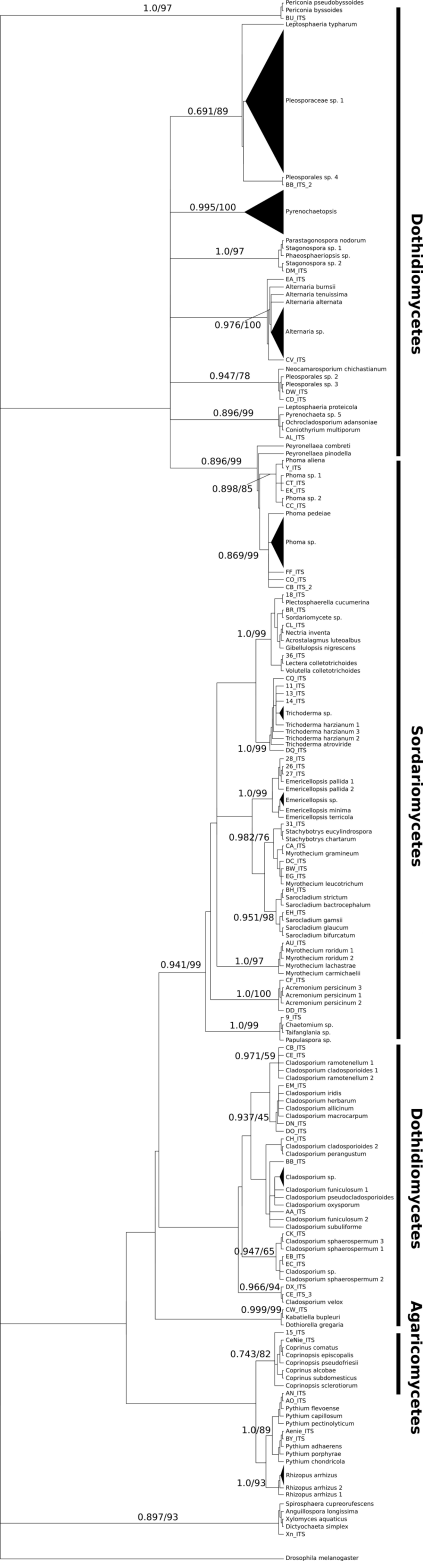
Bayesian tree. Consensus tree of ITS region phylotypes recovered from 126 fungal isolates from water and sediment samples from Cuatro Ciénegas Basin and additional 100 reference sequences from the Genbank, sequence nomenclature corresponds to Table S1. Tree topologies are supported by Bayesian posterior probabilities (first number) and bootstrap values for 1,000 replications (second number). For clarity of the presentation, branches with sequences belonging to the same OTU have been collapsed and are shown as triangles.

**Figure 3:**
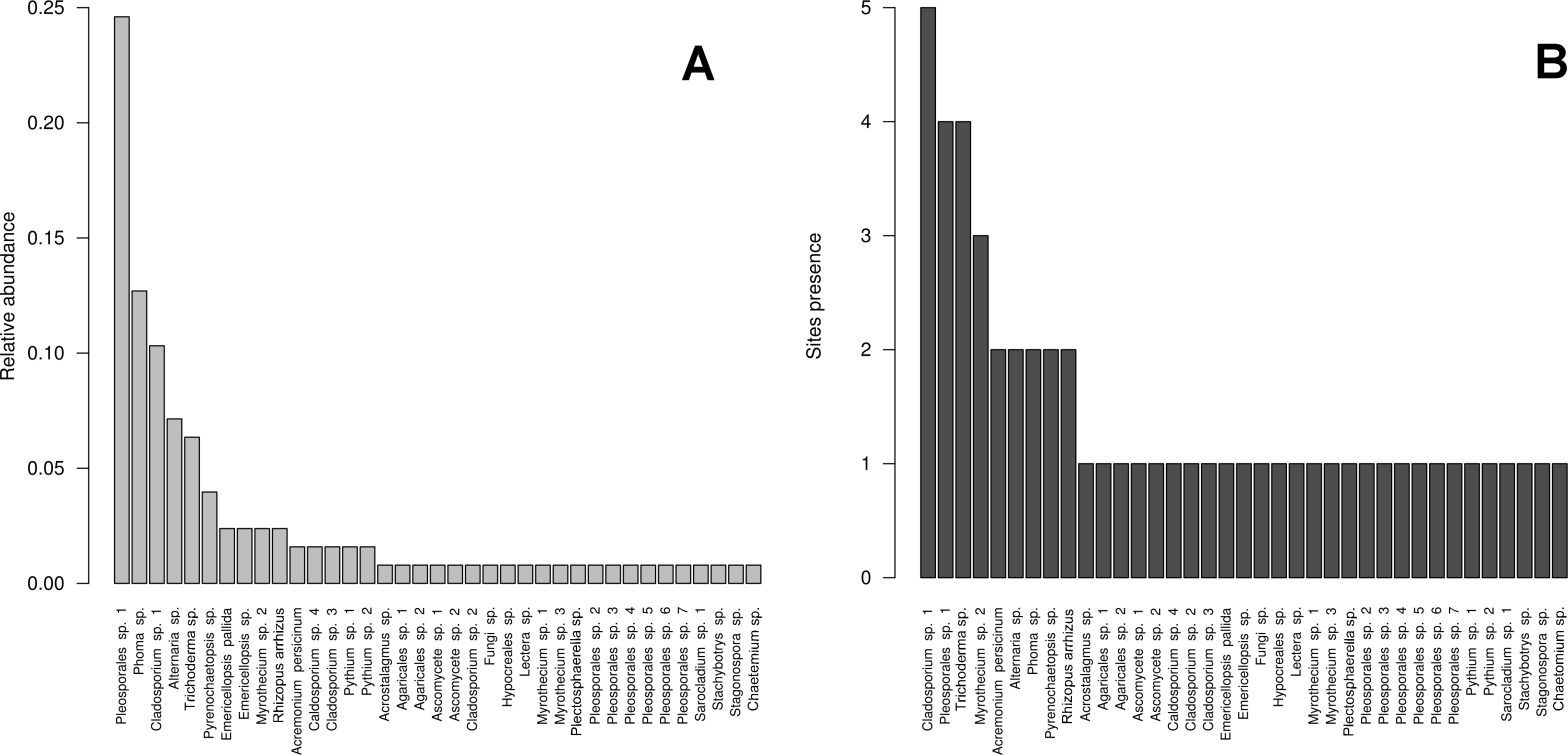
Cultivable OTUs frequency. Microfungal communities inhabiting the studied hydrologic systems in the Cuatro Ciénegas Basin, including cultivable OTUs relative abundance and their distribution in the studied sites. (A) Frequency of the OTUs in the overall sample, and (B) occurrence of the OTUs at a site level.

**Figure 4:**
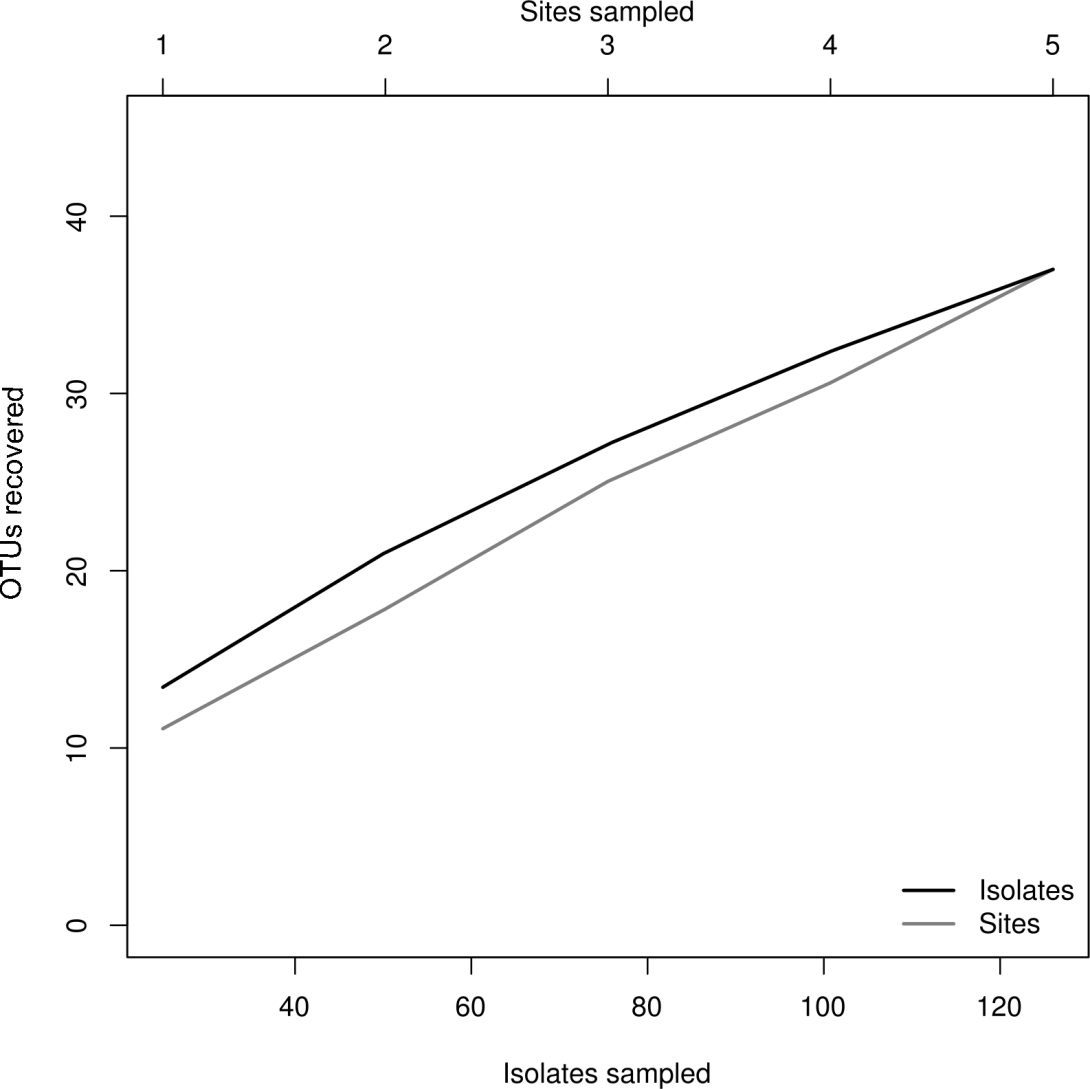
Rarefaction plot. Relationship of sampling effort and OTU richness for cultured fungi from three freshwater systems in the CCB. Dark line indicates the accumulation of taxa as a function of number of isolates, whereas the grey line indicates the accumulation of taxa as a function of number of samples sites.

We isolated a total of 126 pure cultures from sediment and water samples, resulting in 126 assembled sequences ranging from 485 bp to 792 bp of the ITS region, and clustering into 37 OTUs. Taxonomic assignment using the two approaches was consistent. The 37 assigned OTUs were identified up to: kingdom level (1), phylum level (2), order level (10), genus level (21), and species level (3); compring several fungal phyla including Basidiomycota (Agaricales sp. 1, 2), Zygomycota (*Rhizopus arrhizus*), and Ascomycota (31 OTUs). The latest, represented the most abundant and diverse phylum in our samples. The Basidiomycota OTUs were associated to the Agaricomycetes, the Zygomycota OTUs to the Zygomycetes, whereas the Ascomycota to the Sordariomycetes and Dothideomycetes. At an ordinal level, the OTUs were comprised in the *Pleosporales* (11), the *Hypocreales* (11), the *Capnodiales* (4), the *Mucorales* (1), the *Agaricales* (2), and the *Sordariales* (1). The OTUs belonging to *Pleosporales* were the most abundantly isolated, and widely distributed taxa across the samples in different studied sites. Moreover, at a genus level *Cladosporium* and *Phoma* were abundant in the sample as well. Furthermore, our phylogenetic analysis revealed an OTU (Fungus sp.) from CH1 with no taxonomic assignment to major genetic lineages, but clustering within the Pezizomycotina, and 2 OTUs corresponding to the Chromalveolata (Oomycota) genus* Pythium* (Figs. 2 and 3). Moreover, accumulation curve for the isolated ITS region genotypes did not reach an asymptote (Fig. 4).

We obtained a well-supported phylogeny for deeper nodes based on the ITS region data for the 126 cultured isolates from freshwater systems in the CCB. Major lineages of fungi were resolved as monophyletic and with a bootstrap support >70% and Bayesian posterior probabilities >0.7.

**Figure 5:**
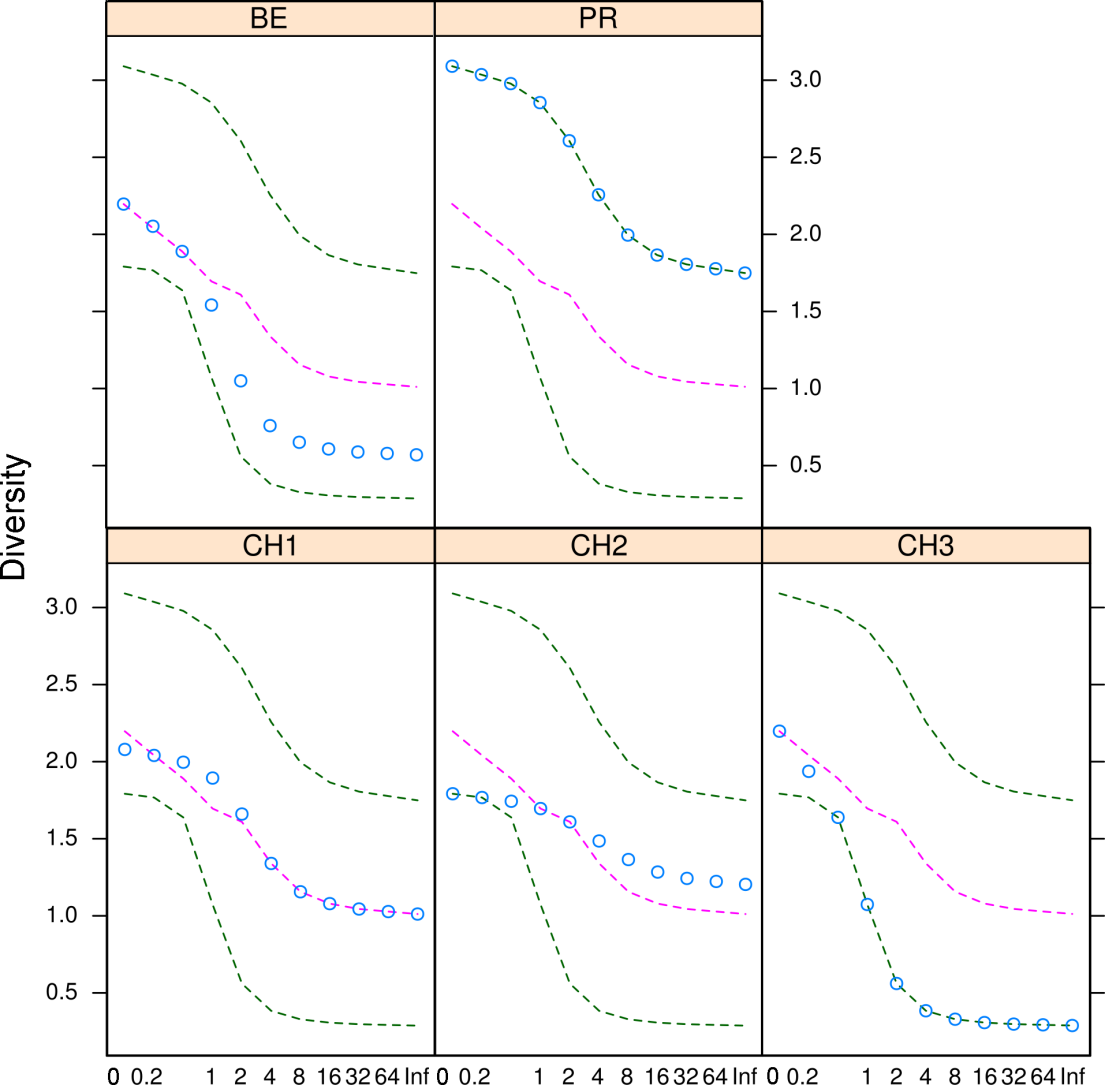
Microfungal diversity in the Cuatro Cienegas Basin. Rényi and Tsallis diversities for each freshwater system. Green dashed lines represent the maximum and minimum diversity values. Pink dashed lines correspond to the median diversity observed for the overall sample. Blue circles indicate the observed diversity values of cultivable fungi in for each system. The *x*-axis refers to the scale used to denote entropy values.

**Figure 6:**
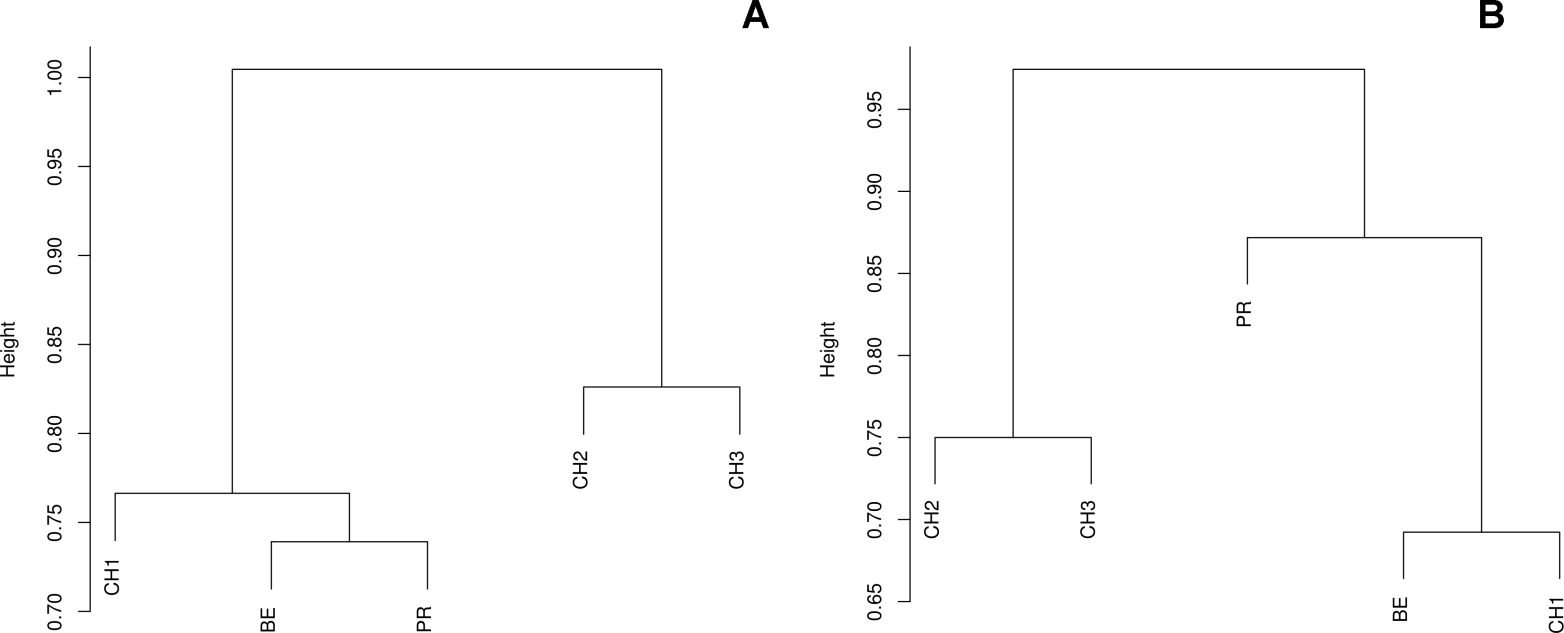
Dissimilarity indexes among sites. Ward’s distance trees based on the (A) Bray-Curtis and (B) Jaccard dissimilarity indexes among the five assessed freshwater systems.

Most of the OTUs correspond to the mitosporic (asexual) stage, comprising several ecological groups (*sensu* Garrett, 1951) commonly obtained from soils, including lignin-decomposing fungi (i.e., *Stachybotrys*), saprotrophic fungi (i.e., *Alternaria*and* Chaetomium*), and plant pathogens (i.e., *Pythium*). We also recorded OTUs with saline affinity (*A. persicinum* and *Emericellopsis pallida*). Moreover, we were able to isolate by culture taxa associated to the genus *Pythium*, although this parasitic Chromista was rare in our samples (Fig. 2).

**Figure 7:**
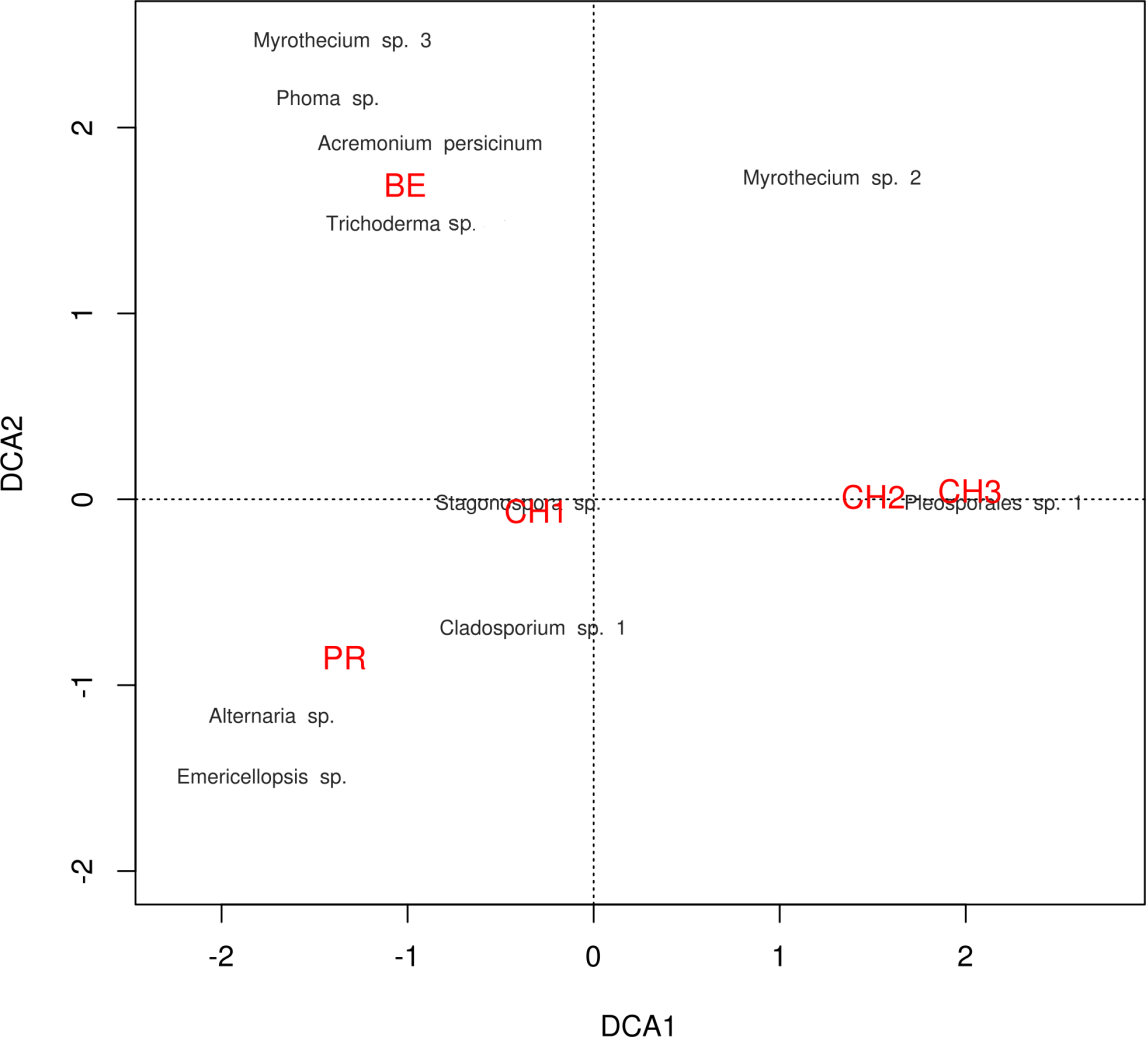
DCA plot. Detrended correspondence analysis of the dominant cultivable microfungal taxa in Cuatro Ciénegas Basin. Dominant OTU names are presented in black, and site names are displayed in red, see Table 1 for nomenclature.

The diversity indexes indicated that PR possess a highest diversity, whereas CH3 presented the lowest values (Fig. S1). Similarly, the *α* parameter of Fisher’s log-series and the Rényi and Tsallis diversity index, resolved that irrespective of sample size, PR has the highest diversity levels; and CH3 the lowest (Table S2, Fig. 5). Moreover, Bray-Curtis and Jaccard dissimilarity indexes indicated that CH2 and CH3 stand as similar systems, whereas CH1 has a distinct species composition (Fig. 6). Dissimilarities in the contribution of OTUs to the overall composition among sites were observed. As the abundant OTUs designated as *Pleosporales* sp. 1 and *Stagonospora* sp. were strongly associated to the Churince system (CH2, CH3 and CH1). Additionally, the OTUs *Acremonium persicinum*, *Trichoderma* sp., *Phoma* sp., and *Myrothecium* sp. 3 occurred preferentially in BE; whereas* Alternaria* and *Emericellopsis* were strongly associated to PR (Fig. 7).

**Figure 8:**
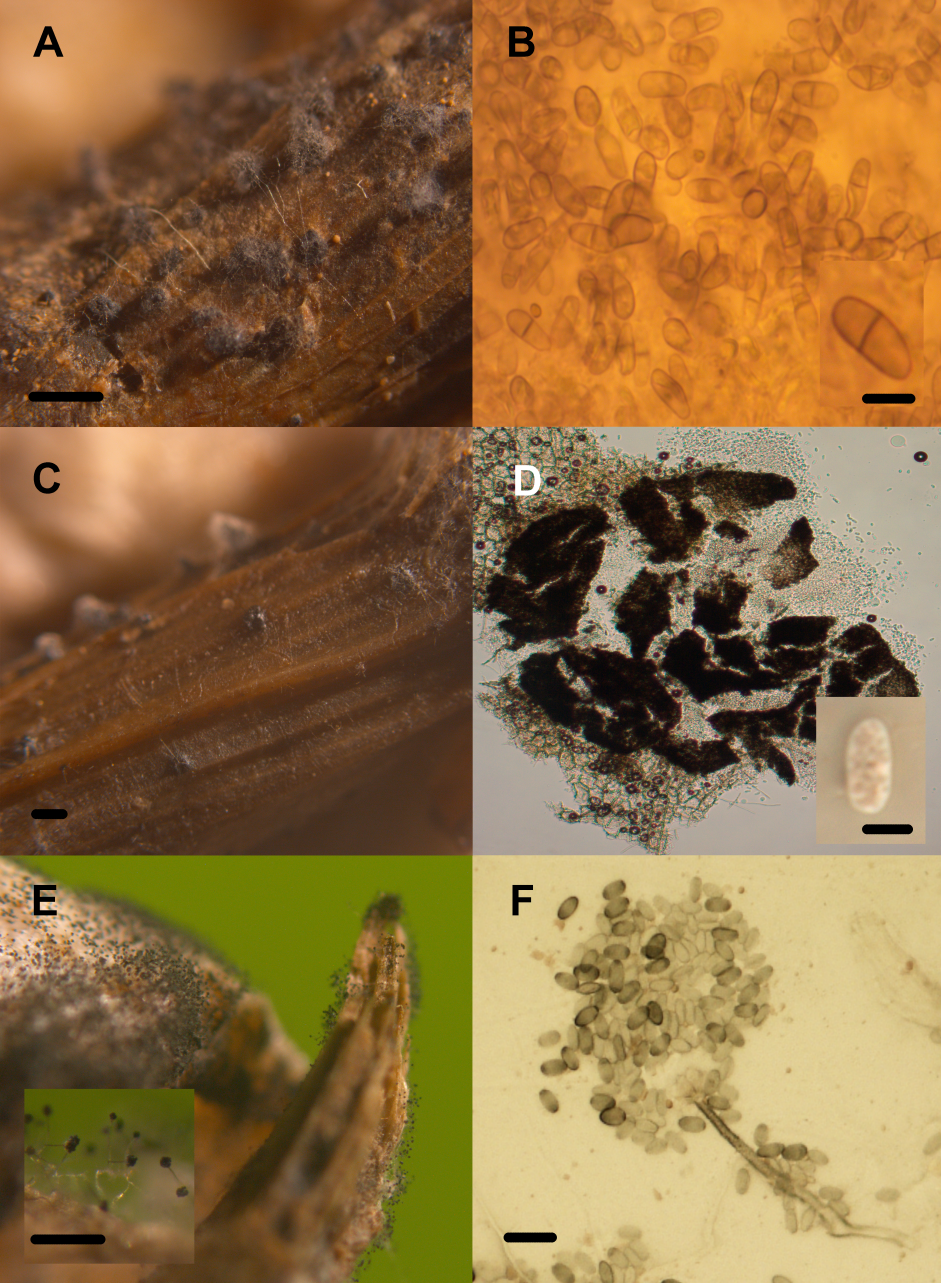
Transient lignocellulolytic aquatic fungi. Diversity of lignocellulolytic transient aquatic fungi occurring on plant wreckage in some water systems in CCB. *Phoma* sp. 1 (A, B), *Phoma* sp. 2 (C, D), *Stachybotrys* sp. 1 (E), *Stachybotrys* sp. 2 (F). Pilose pycnidia growing on wood (A). Pigmented and generally septated conidia (B). Glabrous pycnidia on wood (C). Hyaline, aseptated conidia (D). Conidiomata on *Phragmites* debris (E). Conidiophore and phialoconidia (F). Scale bar: A = 20 µm, B = 27 µm, C = 20 µm, D = 20 µm, E = 20 µm, F = 20 µm.

**Figure 9:**
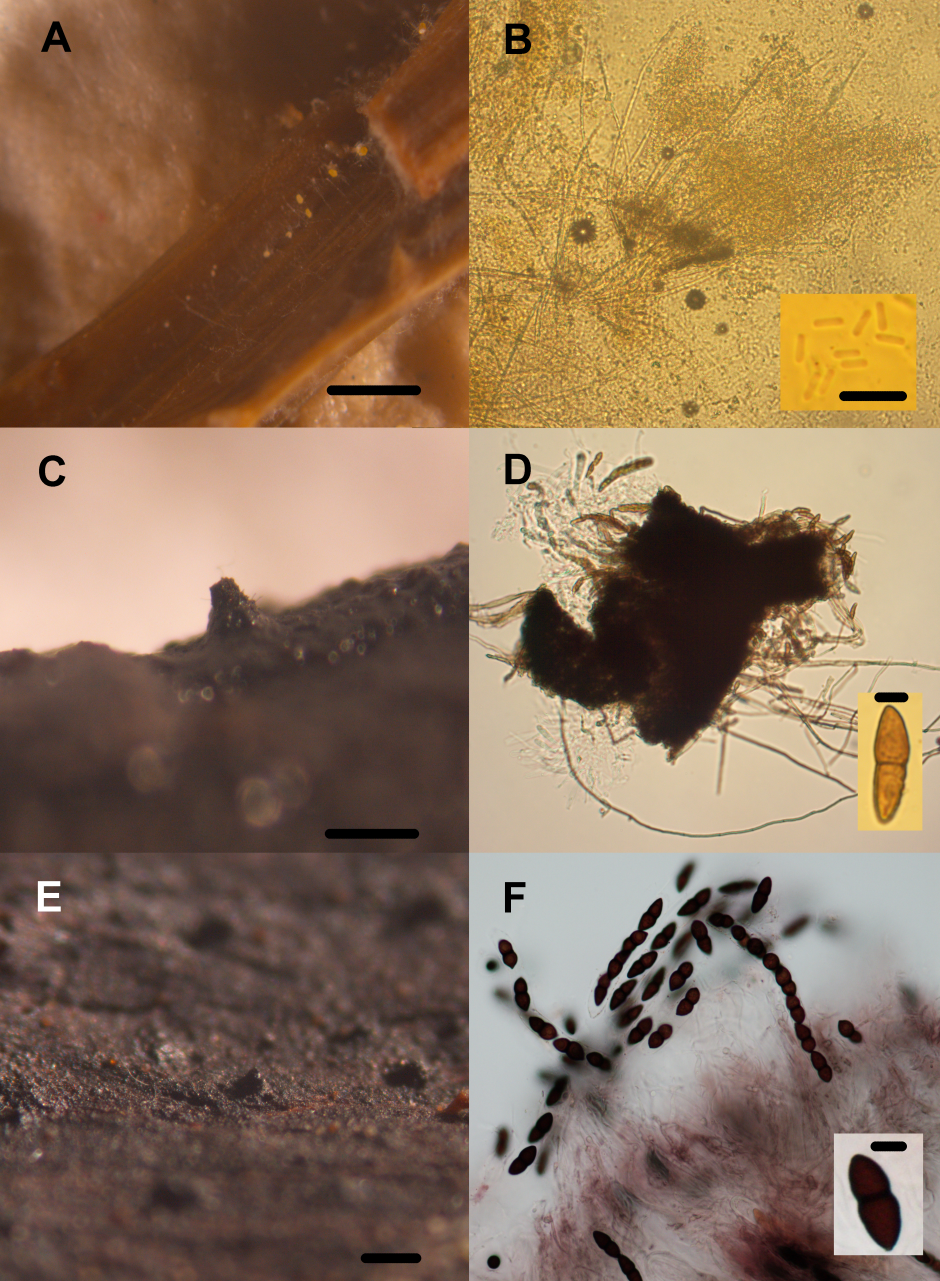
Transient lignocellulolytic aquatic fungi. Diversity of lignocellulolytic transient aquatic fungi occurring on plant wreckage in some water systems in CCB. *Volutella* sp. (A, B), *Venturia* sp. 1 (C, D), *Venturia* sp. 2 (E, F). Sporodochia on substrate (A). Detail of the conidiophores aggregated into sporodochia hyaline with setae around margin of conidiomata, and aseptated hyaline conidia (B). Ascoma erumpent through host bark (C). Squashed subglobose ascoma revealing asci and lightly pigmented 1-septated thick-walled cells (D). Superficial globose ascoma on wood (E). Eight-spored and bitunicated asci, and detail on the morphology of 1-septated, asymmetrical, heavily pigmented thick-walled cells (F). Scale bar: A = 20 µm, B = 20 µm, C = 20 µm, D = 20 µm, E = 20 µm, F = 20 µm.

### Local adaptation to lignocellulose source

Only three panels harbored microfungal structures. Contrastingly, all the autochthonous plant remains were colonized by fungi (Table 2, Figs. 8 and 9). We documented an overall diversity of nine ascomycetes occurring on lignocellulose remains. Two species were recorded in the meiosporic (sexual) stage, and seven in the mitosporic stage. Remarkably, although wood panels were meticulously washed before incubation, mites and nematodes proliferated in the CH panels, and microbial mat covered panels from PR.

**Table 2:** Lignocellulolytic transient aquatic microfungal communities from several water systems in Cuatro Ciénegas Basin.

Taxa	Site	Substrate	Incubation period (mo)
*Arthrobotrys* sp.	CH1	*Pinus* sp. wood panel	6
*Helminthosporium* sp.	PR	*Pinus* sp. wood panel	2
*Phoma* sp. 1	BE	*Phragmites australis*	5
*Phoma* sp. 2	BE	*Phragmites australis*	5
*Stachybotrys* sp. 1	CH2, BE	*Pinus* sp. wood panel,	2
		*Prosopis glandulosa*	
*Stachybotrys* sp. 2	CH2, BE	*Phragmites australis*	2
*Volutella* sp.	BE	*Phragmites australis*	5
*Venturia* sp. 1	CH3	*Prosopis glandulosa*	5
*Venturia* sp. 2	CH3	*Prosopis glandulosa*	5

**Figure 10:**
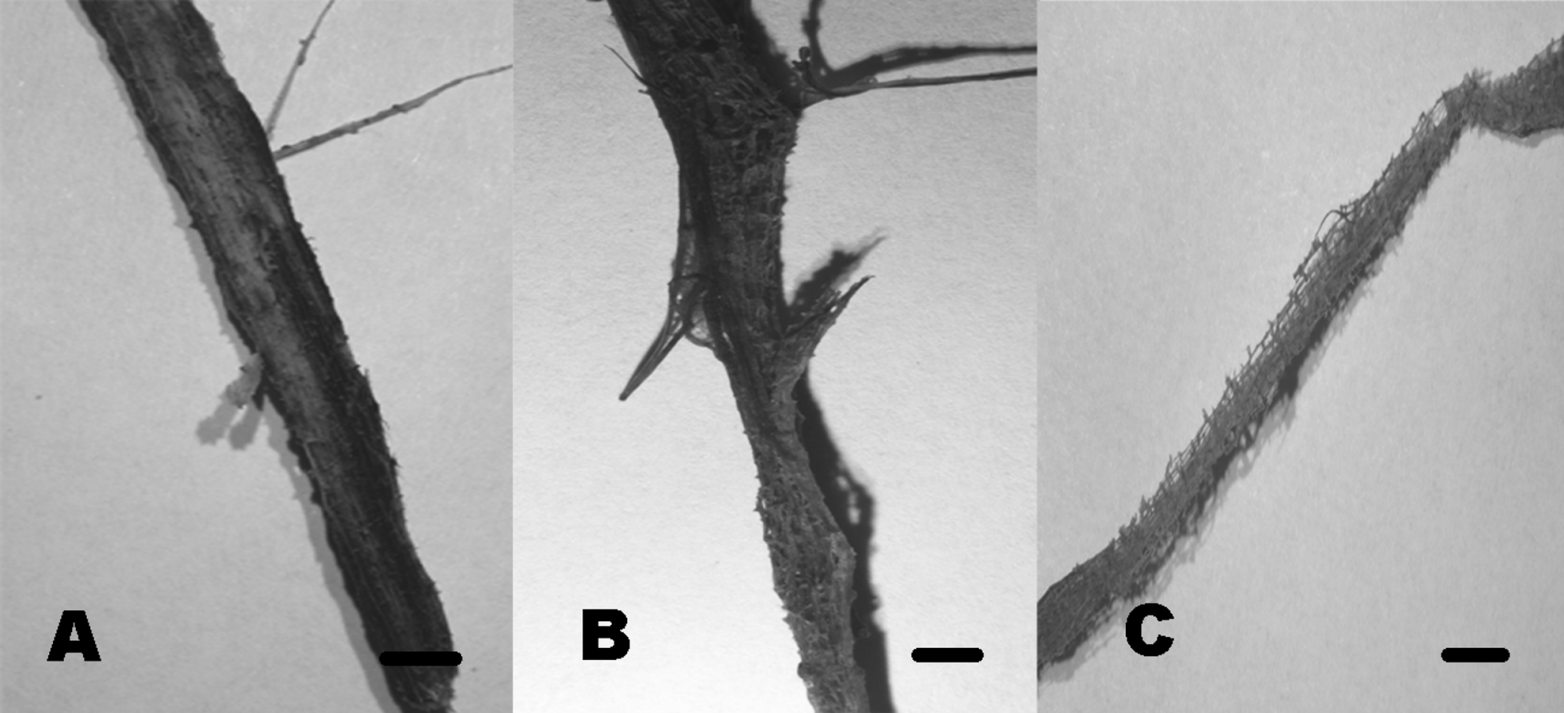
Soft rot cavities produced by lignocellulolytic fungi. Soft rot cavities on local plant wreckage supporting freshwater transient ascomycetes. One-month incubation (A). Three-month incubation (B). Six-month incubation (C). Scale bar = 7 mm

Community composition in test panels and plant remains was similar among the studied sites. However, species richness was higher in BE (six taxa) and CH (five taxa), compared to PR (one taxon). Furthermore, *Volutella* and *Phoma* 1 and 2 strictly occurred in BE, whereas *Arthrobotrys* sp. was only found in CH. We did not observe differential diversity levels among the autochthonous lignicolous and herbaceous substrata, since both plant remains harbored 2–3 taxa. Soft rot decay type was commonly observed in local plant remains, although brown rot was also detected (Fig. 10).

## Discussion

Major lineages of fungi were resolved as monophyletic with high bootstrap support values (≥70%, inferred with ML), resembling the current phylogeny of Ascomycota at a class level clustering the Sordariomycetes, and Dothideomycetes; as well as the Basidiomycota class Agaricomycetes as major lineages (Fig. 2) (Schoch et al., 2009). Although some lineages in the Dothideomycetes were poorly resolved, perhaps as this is the largest and most phylogenetically diverse class within the largest fungal phylum, Ascomycota (Hibbett et al., 2007).

In general, we observed distinct species composition in both samplings, in accordance to the highly fluctuating ecological conditions in the CCB. Though, some frequently isolated OTUs, such as *Cladosporium* sp.1, *Alternaria* sp., *Trichoderma* sp., and *Stachybotrys* sp. were registered in both samplings, suggesting these microfungi represent a permanent component of the mycobiota in the CCB throughout the year. Perhaps these taxa have an important effect on ecosystem processes, conferring short-term resistance to ecosystem function diminutions given by rare species loss (Chapin et al., 2000; Geider et al., 2001; Schwartz et al., 2000). Nevertheless, further studies are needed to formally assess seasonal changes in the microfungal species composition.

### Microfungal diversity

The species accumulation curve indicated that we recorded most of the abundant and common species from the evaluated freshwater systems in the CCB. Moreover, these results suggest that the CCB harbors remarkable fungal diversity levels, agreeing to previous reports accounting the CCB as a biodiversity oasis within the Chihuahuan desert (Souza et al., 2006). Although the number of rare OTUs continued to increase with sampling effort, we obtained a good representation of the microfungal communities, recording taxa from several fungal and non-fungal phyla. Nevertheless, it must be emphasized that it is not possible to accomplish an exhaustive survey using a culture-based approach, since some microorganisms in extreme environments might be uncultivable under the conditions used (Steiman et al., 1997; Oliveira et al., 2013).

The high abundance of cultivable OTUs assigned to the Ascomycota in the microfungal communities in the CCB, agrees with previous reports assessing fungal diversity (Rämä et al., 2014; Millberg, Boberg & Stenlid, 2015). This is not surprising, as the Ascomycota represent the largest phylum of Fungi, with species occurring in numerous ecological niches and virtually all the terrestrial and aquatic ecosystems (Schoch et al., 2009). Our results also indicate that OTUs related to the *Pleosporales* are the most frequently isolated taxa, occurring in all the studied sites. This order comprises a quarter of all dothideomycetous species, and has been recorded from various habitats as epiphytes, endophytes or parasites of living leaves or stems, hyperparasites on fungi or insects, lichenized, or are saprobes of dead plant stems, leaves or bark (Kruys, Eriksson & Wedin, 2006). Similarly, at a genus level *Cladosporium* and *Phoma* were abundant. *Phoma* taxa comprise the most common anamorphs of the *Pleosporales*. These mitosporic fungi may present a saprotrophic stage, but have been mostly reported as pathogenic on herbaceous plants (*Poaceae* and other monocots), which are abundant on the surrounding areas of the studied freshwater systems (De Gruyter et al., 2009; Aveskamp et al., 2010; De Gruyter et al., 2010; Hyde, Mckenzie & Koko, 2011; Zhang et al., 2011). Furthermore, there is strong evidence that *Phoma* isolated from the desert in Mexico play an important role in breaking seed dormancy in the common desert plant *Opuntia streptacantha* (Delgado-Sanches et al., 2011). On the other hand, *Cladosporium* is one of the largest, most heterogeneous and widespread genera of hyphomycetes, including endophytic, fungicolous, human pathogenic, phytopathogenic, and saprobic species (Dugan, Schubert & Braun, 2004).

Although previous studies have suggested that arid saline soils possess no characteristic microfungal diversity, these environments have been commonly associated to osmophilic or halophilic species (Ranzoni, 1968; Moubasher & Mazen, 1972; Moustafa, 1975; Abdel-Fattah, Moubasher & Abdel-Hafez, 1977; Moubasher et al., 1991; Moubasher, 1993; Steiman et al., 1997). Accordingly, we recorded OTUs with saline affinities (i.e., *A. persicinum* and *Emericellopsis pallida*). *Acremonium persicinum* has been formerly isolated from marine sources, whereas *E. pallida* has been evidenced to possess affinity with marine fungal clades (Zuccaro et al., 2004; Suciati et al., 2013). These findings agree with previous reports accounting that the aquatic bacterial diversity in the CCB exhibits ancestral marine affinities (Souza et al., 2006; Alcaraz et al., 2008; Desnues et al., 2008; Moreno-Letelier et al., 2012). Moreover, environmental features of the studied water systems, characterized by high salinity concentrations, might lead to the occurrence and proliferation of fungal species presenting affinity for high osmotic pressures.

The isolated microfungi in our study have the ability to tolerate extreme and nutrient-poor environments, indicating that these taxa possess specific adaptive mechanisms to survive in challenging conditions. These adaptive traits might be linked to morphological features, such as the production of resistant structures, melanized conidia, among others (Gorbushina, 2003). Moreover, these fungi comprise several ecological groups commonly isolated from soils, as air-borne microfungi, including lignin-decomposing fungi, saprotrophic fungi, and plant pathogens, comprising a high functional fungal diversity, perhaps due to high spatial and temporal heterogeneity, as predicted by the intermediate disturbance hypothesis for desert ecosystems (Connell, 1978; Zak, Sinsabaugh & Mackay, 1995).

Although P limitation to primary productivity and other biological processes is a well-known phenomenon (Vitousek et al., 2010), information about its effect on fungal communities is still rare. Previous surveys on soil mycobiota indicate that fungal growth may be limited when nutrient availability falls below a certain threshold (Treseder & Allen, 2002). Besides, experimental data on low nutrient aquatic systems suggest that saprotrophic fungi acquire and use nutrients from both the plant wreckage and the surrounding water (Grattan & Suberkropp, 2001). Our results resemble these findings, since most of the obtained cultivated OTUs, as well as lignocellulolytic taxa from wood baits are transient saprotrophic taxa. As water systems in the CCB are highly oligotrophic, the aquatic mycobiota perhaps transits from water to terrestrial environments in order to uptake limiting essential nutrients. This strategy might allow fungi to develop under the most suitable conditions. Conclusively, the analyzed fungi in this study have a wide tolerance to P depletion, presenting different P requirements compared to fungi inhabiting non-oligotrophic sites.

Numerous indexes combining measures of richness and abundance have been proposed in order to fully describe the biodiversity in natural communities. Hence, to obtain a better understanding and describe different features of cultivable microfungal communities in the CCB, we calculated various diversity indexes. Our findings indicated that microfungal communities in our samples from the CCB are characterized by few dominant OTUs, some common taxa and several rare OTUs. Our results on the low diversity values in BE, plus the variable levels of diversity, and patchy distribution of microfungal communities within the Churince hydrologic system (CH1, CH2 and CH3) agree with previous studies on the diversity of aquatic prokaryotic communities (Escalante et al., 2008). The diversity patterns presented here also agree with previous findings describing low local diversity levels, but marked differences in diversity among sites (Escalante et al., 2008). Hence, our first hypothesis suggesting low local diversity perhaps due to the low nutrient and harsh environmental conditions inherent to deserts was confirmed.

Mycological studies on desert soil are rather limited. Several authors assume the diversity of fungi is low compared to tropical regions (Sterflinger, Tesei & Zakharova, 2012). Globally, our results indicated that the CCB possess a moderate taxonomical diversity compared to other oasis and semi-arid environments. Yet vegetal richness in these systems is higher compared to the CCB, perhaps leading to a higher fungal diversification as proposed by Hawksworth (2001). Nevertheless, compared to dry systems in the Antarctica and Namibia, microfungal diversity in the CCB is remarkable (Bridge & Newsham, 2009; Ciccarone & Rambelli, 1998; Oliveira et al., 2013; Rao et al., 2012; Steiman et al., 1997).

We recorded several plant pathogens and strong lignocellulolytic OTUs (i.e., *Alternaria*, *Cladosporium, Stachybotrys* and *Trichoderma*), which have been reported earlier from desert soils (Abdel-Hafez, 1982a; Guiraud et al., 1995; Steiman et al., 1995). These taxa possess a worldwide distribution, suggesting that certain cosmopolitan soil fungi are able to adapt to extreme life conditions such as high osmolarity or elevated temperatures (Moustafa, 1975; Abdel-Hafez, 1982b; Abdullah & Zora, 1993). However, our results disagree with further culture-dependent studies reporting *Aspergillus*, *Ulocladium*, *Penicillium*, *Ascobolus*, *Periconia* to yeasts including the genera *Debaryomyces*, *Candida* and *Cryptococcus* as an abundant component of microfungal communities in arid environments (Abdel-Hafez, 1982a; Arenz & Blachette, 2011; Conley et al., 2006; El-Said & Saleem, 2008). Nevertheless, it has been documented that in response to the high temporal and spatial variability, desert organisms tend to be functionally diverse, ensuring quick adaptation to favorable conditions (Gochenaur, 1975). Therefore, discrepancies among the fungal community structures in diverse arid environments might be reasonable.

### Geographic differentiation

Community dissimilarity indexes suggested that the Churince is a heterogeneous hydrological system, as CH1 is divergent from CH2 and CH3, despite the fact that these neighboring sites are less than 800 m from each other. These results resemble the hydrological features of the Churince system, where differentiation might be associated to ecosystemic zonification (lentic and lotic areas), geographic proximity, the composition of contiguous vegetation, and the direction of water flow.

Moreover, the Bray-Curtis index indicated that CH1, BE and PR share the occurrence of dominant OTUs. However, the Jaccard index indicated that PR is highly differentiated in terms of community composition independently from the abundance of OTUs. Similarities on the cultivable fungal community structure of CH and BE agree with environmental features, such as the high levels of sulfates in both systems. This findings also match previous work reporting a high similarity between the prokaryotic communities of BE and CH perhaps as a result of underground water connectivity (Escalante et al., 2009). Likewise, the high divergence of PR might be associated to the totally distinctive conditions of this system, where microbial diversity experience highly variable conditions (Rebollar et al., 2012; Souza et al., 2012).

Likewise, through the DCA we identified dissimilarities in terms of the relative contribution of cultivable OTUs to the overall differences among sites, with the abundant occurrence of *Pleosporales* sp. 1 in CH2 and CH3, *Stagonospora* sp. in CH1, *Alternaria* and *Emericellopsis*in PR, and *A. persicinum*, *Trichoderma* sp., *Phoma* sp., and *Myrothecium* sp. 3 in BE. These results confirmed further assumptions about microfungi presenting similar geographical patterns to bacteria and viruses where few dominant taxa have a wide distribution, and rare taxa are private to a single site (Souza et al., 2012; Desnues et al., 2008).

### Local Adaptation to lignocellulolytic source

A central concern for microbial ecology is to unveil the role that microbes play in the biogeochemical cycling, particularly in light of the interest over the potential use of fungal enzymes (Smedsgaard & Nielsen, 2005). However, previous studies on the microfungal diversity in arid environments have employed cultivation and environmental DNA approaches, though these methodologies do not reveal whether fungi play an active ecological role in the degradation of plant remains (Rao, Hyde & Pointing, 2013). Remarkably, by using a moist chamber approach, we were able to demonstrate that the recorded microfungi actively contribute to the preferential decomposition of native lignocellulolytic substrates in CCB, since most of the taxa were obtained from autochthonous plant remains. Our findings suggest lignocellulolytic fungi in the CCB are highly adapted to rapidly decompose local organic matter in response to interspecific competition among the entire microbial community. Furthermore, the differential chemical composition in terms of the lignin, hemicellulose, and cellulose content in the test blocks compared to the natural occurring plant remains might also have affected the occurrence of lignocellulolytic fungi, since these microorganisms might be adapted to preferably consume local plant remains.

Our results on the low microfungal diversity occurring on lignocellulose materials might be related to the short and sporadic rainy season and high temperatures restricting fungal development, leading to a lack of turnover in the dominant fungal species; coupled with a possible strong antagonism among fungi and bacteria (containing a large number of genes related to the production and resistance to antibiotics) given by substrate competition, and a possible tradeoff between fungal growth and tolerance towards bacteria (Zak, Sinsabaugh & Mackay, 1995; Breitbart, Hoare & Nitti, 2009; Bonilla-Rosso et al., 2012; Peimbert et al., 2012; Ponce-Soto et al., 2015).

Plant litter decomposition in arid environments may occur via photodegradation, microarthropods, fungi or bacteria (Whitford et al., 1986; Austin & Vivanco, 2006). Bacterial wood decay in freshwater tends to be superficial compared to fungal attack, concluding that the role of bacteria is less important (Holt & Jones, 1983; Eslyn & Moore, 1984; Singh & Butcher, 1991). Although prokaryotic communities in CCB have been acknowledged to notably contribute to ecosystem processes, we confirmed that microfungi also represent key elements in the wood decay within the freshwater springs (Bucher et al., 2004). Microfungal taxa were observed acting along with microarthropods and bacteria in the deterioration of the test wood panels, chiefly producing soft rot (Simonis, Raja & Shearer, 2008). To our knowledge, this work represents the first analysis on the diversity of lignocellulolytic microfungi occurring in an oligotrophic desert ecosystem.

Finding exclusively members of the Ascomycota agrees with preceding investigations reporting few freshwater basidiomycetes from freshwater samples, and none being aggressive wood decay fungi (Eaton & Hale, 1993; Hyde & Goh, 1998). Moreover, all the documented microfungi belong to the freshwater transient ecological group, whereas resident aquatic fungi were not recorded. The obtained freshwater transient fungi included saprobiotic taxa previously reported from soil, leaf litter, wood remains, as well as nematode predatory fungi. These findings indicate that the freshwater transient ecological group of microfungi might be well adapted to long-term biotic and abiotic stress, as resident aquatic fungi have been reported to be unsuccessful under stress conditions (Luo et al., 2004).

The most common lignocellulolytic ascomycete identified was *Stachybotrys* sp. 2, often occurring on leaf remains of the perennial wetland grass *Phragmites australis*. Species comprised within this anamorphic genus have been reported to be strong cellulose degrading fungi, occurring as saprobes in soil or on litter and occasionally as animal pathogens (Wang et al., 2015). Additionally, taxa belonging to the genera *Venturiaceae*, have a parasitic or saprobic lifestyle, occurring on leaves or stems of dicotyledons. *Volutella* is a common soil hyphomycete genus that has received little study, despite the common occurrence and broad distribution. *Phoma* represents a widely distributed genus occurring in soil, including saprobes and plant pathogenic species (Tokumasu & Aoiki, 2002; Boerema et al., 2004; Gräfenhan et al., 2011; Zhang et al., 2011). The occurrence of these fungi under environmental stress, and strong competition conditions might imply they are highly adapted to cope with the local microbial community, representing strong and highly adapted competitors and predators (Barron, 2003).

Community composition of lignocellulolytic microfungi was similar among the studied sites. However, registering a higher taxonomic diversity in BE and CH compared to PR might be related to the hydrologic connection between BE and CH systems, which has been previously proposed based on the analysis of the prokaryotic communities (Escalante et al., 2009; Bonilla-Rosso et al., 2012; Peimbert et al., 2012). Additionally, the proliferation of bacteria on test panels in PR denotes the strong competition for substrata among the microbial community, increasing the selective pressure in this site and lowering the microfungal diversity (Ponce-Soto et al., 2015).

Our results on the dominance of mitosporic over meiosporic taxa on lignocellulose remains and on the cultivable OTUs from water and sediment samples, agree with previous studies on the microfungal diversity in arid and semiarid areas, where mitosporic species dominate over meiosporic species (Abdullah & Al-Bader, 1990; Mouchacca, 2005; Oliveira et al., 2013). The dominance of mitosporic taxa in arid regions might be related to their adaptability to stress conditions, whereas sexual ascomycetes are believed to be stress-selected. The absence of a meiosporic stage has been recognized as a factor involved in the stress adaptation of fungi (Guiraud et al., 1995; Steiman et al., 1995; Sterflinger, Tesei & Zakharova, 2012). This may suggest that mitosporic evolutionary linages are successful over meiosporic linages in adapting to this extreme habitat.

## Conclusions

Here we present the first analysis of the microfungal communities inhabiting freshwater systems in the oligotrophic desert oasis of CCB. We recorded cultivable fungal OTUs with saline affinities, as well as several plant pathogens and lignocellulolytic OTUs with a worldwide distribution, suggesting these microfungi are able to adapt to extreme life conditions. Moreover, our findings suggest that the lignocellulolytic microfungal communities are dominated by few anamorphic transient taxa adapted to preferably breakdown local plant remains resisting the strong inter- and intraspecific competition. As predicted we recorded a moderate microfungal diversity compared to other arid environments, perhaps due to strong selective conditions such as environmental oligotrophy, and antagonistic interactions with the highly competitive prokaryotic community. Additionally, we did not observe a clear geographic differentiation between the study sites; nevertheless the dissimilarity indexes indicated that the Churince hydrological system is heterogeneous, and that PR is differentiated in terms of community composition independently from the abundance of OTUs. However, samples from CH1, BE and PR share the occurrence of abundant OTUs. Lastly, we documented the occurrence of lignocellulolytic microfungi on local vegetation remains compared to *Pinus* wood traps, possibly as a result of a high specificity and adaptation to the local lignocellulose source.

Recently there is a growing interest in describing life in extreme environments, due to its economic potential for biotechnological applications (Rothschild & Mancinelli, 2001). Particularly, fungi have been proved to possess a long-term ecological activity during drying cycles in arid ecosystems compared to other soil microorganisms (Freckman, 1986). Here we present the first analysis of the microfungal communities inhabiting freshwater systems in the ultra-oligotrophic desert oasis of CCB.

Assessing the fungal diversity occurring in ultra-oligotrophic freshwater systems is important, since this ecological group of microorganisms plays a key ecological role, and represents an important indicator of trophic complexity and biotic interactions among microbial communities. Our results set the basis to unveil the ecological role of fungi growing under long-term environmental stress conditions. However, further studies are needed, particularly on the interactions between fungi and bacteria, and the ecological stoichiometry of fungi to fully understand the eukaryotic survival at the ultra-oligotrophic and arid limit for life.

## Supplemental Information

10.7717/peerj.2064/supp-1 Figure S1 Diversity values of cultivable fungi obtained for each of the five freshwater systems in the Cuatro Ciénegas Basin Site abbreviations as in Table 1. *H*′ is equally sensitive to rare and abundant species and increases as both the richness and the evenness of the community increase. Whereas *D*, is a complement of *H*′ representing the probability that two randomly chosen individuals belong to different species, and is heavily weighted towards most abundant species. *J* and *E* represent evenness estimates indicating the degree to which individuals are split among species, where low values indicate that one or a few species dominate (Dejong, 1975).

10.7717/peerj.2064/supp-2 Table S1 NCBI sequence accession numbers

10.7717/peerj.2064/supp-3 Table S2 Diversity estimates

## Additional Information and Declarations

### Field Study Permissions

The following information was supplied relating to field study approvals (i.e., approving body and any reference numbers): Secretaría de Medio Ambiente y Recursos Naturales, Subsecretaría de Gestión para la protección Ambiental, Dirección de Vida Silvestre FAUT-0230.

### DNA Deposition

The following information was supplied regarding the deposition of DNA sequences: Sequences were deposited (submission ID: 1899023) in the GenBank database. In Table S1 we included the NCBI accession number for all our sequences.
