# Supplementary figures and images for: Microfungal oasis in an oligotrophic desert: diversity patterns and community structure in three freshwater systems of Cuatro Ciénegas, Mexico
Source: PeerJ. 2016 Jun 2;4:e2064. doi: 10.7717/peerj.2064 (PMC4893334; doi:10.7717/peerj.2064)

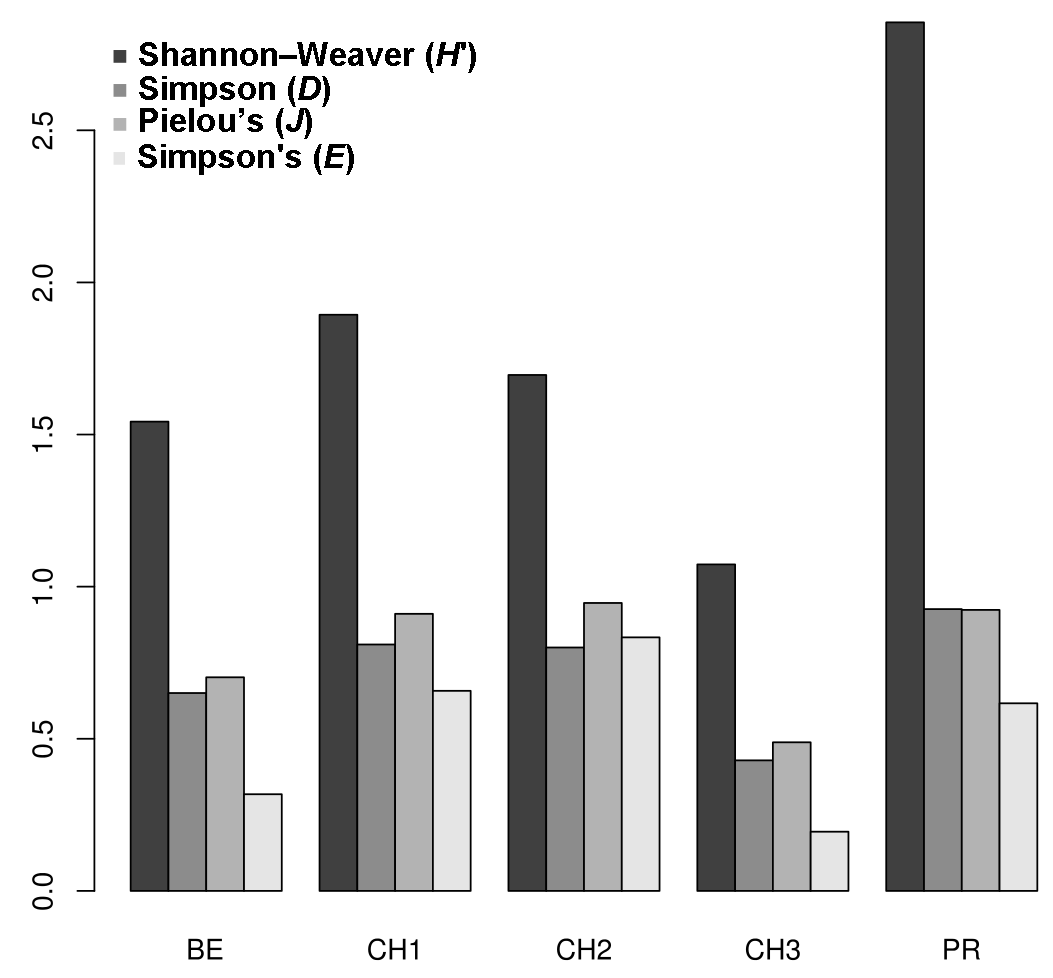

Supplement: Figure S1 — Site abbreviations as in Table 1. H′ is equally sensitive to rare and abundant species and increases as both the richness and the evenness of the community increase. Whereas D, is a complement of H′ representing the probability that two randomly chosen individuals belong to different species, and is heavily weighted towards most abundant species. J and E represent evenness estimates indicating the degree to which individuals are split among species, where low values indicate that one or a few species dominate (Dejong, 1975). [file peerj-04-2064-s001.png]
